# Kynurenic Acid Protects Against Myocardial Ischemia/Reperfusion Injury by Activating GPR35 Receptors and Preserving Mitochondrial Structure and Function

**DOI:** 10.3390/biom15101481

**Published:** 2025-10-21

**Authors:** Dóra Nógrádi-Halmi, Barbara Erdélyi-Furka, Dóra Csóré, Éva Plechl, Nóra Igaz, László Juhász, Marietta Zita Poles, Bernát Nógrádi, Roland Patai, Tamás Ferenc Polgár, Mónika Kiricsi, László Vécsei, Renáta Gáspár, Tamás Csont

**Affiliations:** 1 Department of Biochemistry, Albert Szent-Györgyi Medical School, University of Szeged, H-6720 Szeged, Hungary; halmi.dora@med.u-szeged.hu (D.N.-H.); erdelyi.furka.barbara@med.u-szeged.hu (B.E.-F.); csore.dora@med.u-szeged.hu (D.C.); plechl.eva@med.u-szeged.hu (É.P.); 2 Interdisciplinary Centre of Excellence, University of Szeged, H-6720 Szeged, Hungary; 3 Division of Haematology, Department of Internal Medicine, Albert Szent-Györgyi Clinical Center, University of Szeged, H-6720 Szeged, Hungary; 4 Doctoral School of Multidisciplinary Medical Sciences, University of Szeged, H-6720 Szeged, Hungary; 5 Doctoral School of Experimental and Preventive Medicine, University of Szeged, H-6720 Szeged, Hungary; polgar.tamas.ferenc@gmail.com; 6 Department of Biochemistry and Molecular Biology, Faculty of Science and Informatics, University of Szeged, H-6720 Szeged, Hungary; noraigaz@gmail.com (N.I.); kiricsim@gmail.com (M.K.); 7 Institute of Surgical Research, Albert Szent-Györgyi Medical School, University of Szeged, H-6720 Szeged, Hungary; juhasz.laszlo.1@med.u-szeged.hu (L.J.); poles.marietta.zita@med.u-szeged.hu (M.Z.P.); 8 Institute of Biophysics, Biological Research Centre, Hungarian Research Network, H-6720 Szeged, Hungary; bernatnogradi@gmail.com (B.N.); roland.patai.1988@gmail.com (R.P.); 9 Department of Neurology, Albert Szent-Györgyi Health Centre, University of Szeged, H-6720 Szeged, Hungary; vecsei.laszlo@med.u-szeged.hu; 10 HUN-REN-SZTE Neuroscience Research Group, University of Szeged, H-6720 Szeged, Hungary

**Keywords:** kynurenic acid, Trp metabolites, ischemia/reperfusion injury, mitoprotection

## Abstract

Acute myocardial infarction, often associated with ischemia/reperfusion injury (I/R), is a major healthcare issue ranking among the leading causes of death globally. Although kynurenic acid (KYNA), an endogenous tryptophan metabolite, has been previously shown to protect the cardiac tissue against I/R injury, its mechanism of action remains unclear. Therefore, here, we examined whether KYNA administration rescues H9c2 cardiac cells exposed to I/R through the preservation of the structural and functional integrity of the mitochondria. In addition, we assessed whether KYNA-derived agonism on G-protein coupled receptor 35 (GPR35) is involved in the protection of cardiac cells against simulated I/R (SI/R)-induced cellular demise. Our results demonstrated that KYNA attenuated the SI/R-induced calcium overload as well as impairments in the mitochondrial ultrastructure. Furthermore, administration of KYNA was shown to reduce mitochondrial superoxide production and preserve mitochondrial function in cells exposed to SI/R. Activation of the GPR35 receptors using an agonist other than KYNA rescued cardiac cells undergoing SI/R, attenuated the apoptotic activity, and improved various parameters of mitochondrial respiration. The administration of a synthetic GPR35 antagonist in parallel with KYNA attenuated the KYNA-induced cytoprotection. Our findings provide evidence that the protective effect of KYNA against SI/R-induced cardiac cell injury involves mitoprotective mechanisms, acting, at least in part, through the activation of GPR35 receptors.

## 1. Introduction

Ischemia/reperfusion (I/R)-associated cardiovascular diseases, including acute myocardial infarction (AMI), represent a significant healthcare challenge, ranking among the leading causes of mortality worldwide. The underlying myocardial ischemia arises from severe impairment of the coronary circulation, causing an imbalance between oxygen and nutrient supply and demand, which eventually culminates in irreversible cardiomyocyte demise [1]. In recent decades, early recanalization of the cardiac arteries and subsequent reperfusion of the myocardium have been established as a primary approach to salvaging cardiac muscle undergoing ischemia [2]. Nevertheless, a considerable amount of literature has been published on the harmful effects of revascularization, describing that reperfusion may evoke further myocardial injury, often referred to as ‘reperfusion injury’ [3]. The pathomechanism of I/R-induced cardiac cell damage comprises several mechanisms, including, but not limited to, increased oxidative stress and impairment of the structural and functional integrity of the mitochondria, as well as activation of various cell death pathways (e.g., necrosis, necroptosis, apoptosis, and autophagy-dependent cell death) [4]. Among these, mitochondrial impairment is thought to be the centerpiece of I/R-related cardiac pathologies, laying behind decreased production of ATP and the accumulation of various toxic compounds (i.e., lactate buildup, increased levels of reactive oxygen and nitrogen species, calcium overload, etc.), as well as cytochrome C release and subsequent activation of programmed cell death, leading to the loss of cardiomyocytes [5]. Due to its significant contribution to the development of myocardial I/R-injury, preservation of mitochondrial integrity seems to be a promising approach to enhancing cardiac tolerance against an I/R insult.

Kynurenic acid (KYNA) is a natural compound synthesized endogenously during the degradation of tryptophan through the kynurenine pathway [6]. It has been shown to exert antagonistic effects on various ionotropic glutamate receptors (i.e., N-methyl-D-aspartate (NMDA), α-amino-3-hydroxy-5-methyl-4-isoxazolepropionic acid (AMPA), and kainate receptors) and stimulate G-protein coupled receptor 35 (GPR35) and aryl hydrocarbon receptors (AhRs) [7]. KYNA-derived effects have been extensively studied in the field of neurology over the past decades, revealing that it shows neuroprotective properties in various neurodegenerative diseases, as well as I/R-induced injuries of the nervous tissue [8,9,10,11], which have mostly been attributed to its NMDA-receptor-antagonistic features [12]. However, several investigations have been carried out recently to analyze its potential effects derived through the modulation of other target receptors, as well as to assess whether KYNA mediates protective mechanisms outside of the nervous system too. These studies revealed that KYNA-induced GPR35 receptor agonism reduces inflammasome activation through the amelioration of calcium mobilization [13], improves energy metabolism [14,15], and shows anti-inflammatory, immunosuppressive [16], and antinociceptive [17] effects. Further supporting its beneficial role outside of the nervous system, KYNA has been shown to exert protection against intestinal [18], endometrial [19], and liver injuries [20,21]. Importantly, its beneficial effects on I/R-induced pathologies have been confirmed outside of the nervous system too. Olenchock et al. reported that KYNA plays a fundamental role in the mediation of the cardioprotective effect of remote ischemic conditioning [22]. Recently, we have corroborated these promising effects, revealing that KYNA-derived cardiocytoprotection involves antiapoptotic properties [23]. Interestingly, in the setting of myocardial I/R, the NMDA-receptor-antagonistic feature of KYNA did not seem to contribute to its protective effect [23]. Nevertheless, GPR35 receptors are another promising mediator potentially involved in the mechanism of KYNA-induced cytoprotection.

In the present study, we aimed to further elaborate on the KYNA-induced preservation of mitochondrial integrity, as well as to characterize the role of GPR35 agonism in the setting of cardiac I/R injury.

## 2. Materials and Methods

### 2.1. H9c2 Cell Culture

H9c2 rat cardiomyoblasts (ATCC; Manassas, VA, USA; RRID: CVCL_0286) were cultured in Dulbecco’s modified Eagle’s medium (DMEM; Biosera, Cholet, France; Cat#LM D1108) supplemented with 10% *v*/*v* fetal bovine serum (FBS; Biosera; Cholet, France; Cat#FB-1090), 200 nM glutamine (Gibco, Grand Island, NY, USA; Cat#25030024), and 1% *v*/*v* antibiotic/antimycotic cocktail (Biosera; Cholet, France; Cat#XC-A4110). Cells were cultured in 25 cm^2^ and 75 cm^2^ tissue culture flasks and were subcultured each time they reached 70–80% confluence. Cells were seeded at a density of 4.5 × 10^3^ cells/well into 96-well plates for viability measurements and 2 × 10^4^ cells/well into 24-well plates containing glass coverslips for MitoTracking, mitochondrial membrane potential, and superoxide production measurements. To collect samples for Western blotting, real-time polymerase chain reaction (qPCR), electron microscopy, and high-resolution respirometry, cells were grown in 75 cm^2^ tissue culture flasks. Two days after seeding, the cells were exposed to the different experimental protocols.

### 2.2. Experimental Design

#### 2.2.1. Simulated Ischemia/Reperfusion

Simulated ischemia/reperfusion has been performed in vitro, as described previously [23,24]. Briefly, H9c2 cardiomyoblasts were subjected to 6 h of simulated ischemia, followed by 2 h of simulated reperfusion. During simulated ischemia, cells were kept in a glucose-free hypoxic solution (containing 119 mM NaCl, 5.4 mM KCl, 1.2 mM NaH_2_PO_4_, 0.5 mM MgCl_2_, 5 mM HEPES, 1.3 mM MgSO_4_, 0.9 mM CaCl_2_, 20 mM Na-lactate, and 0.1% BSA, pH 6.4) and placed into a hypoxic incubator (Panasonic MCO-5M-PE O_2_/CO_2_ incubator, PHCbi Europe, Breda, Netherlands; 0.4% O_2_; 95% N_2_, 5% CO_2_, 37 °C) in order to maintain ischemic conditions. Next, hypoxic solution was replaced with 10% FBS-containing media supplemented with the corresponding treatments, and cell cultures were placed into a standard CO_2_ incubator (MCO-170AIC-PE CO_2_ incubator, PHCbi Europe, Breda, The Netherlands; 5% CO_2_, 37 °C) for 2 h to mimic reperfusion. A control group of cells was kept under normoxic conditions throughout the protocol. Control cells were maintained in a standard CO_2_ incubator, in normoxic solution (containing 125 mM NaCl, 5.4 mM KCl, 1.2 mM NaH_2_PO_4_, 0.5 mM MgCl_2_, 20 mM HEPES, 1.3 mM MgSO_4_, 1 mM CaCl_2_, 15 mM glucose, 5 mM taurine, 2.5 mM creatine-monohydrate, and 0.1% BSA 0.1%, pH 7.4) and media, in parallel with simulated ischemia and reoxygenation, respectively.

#### 2.2.2. KYNA Treatment

KYNA treatment was administered during the entirety of the SI/R at the previously uncovered protective concentration of 64 µM [23]. To provide treatment solutions, a 10 mM stock solution was prepared by dissolving KYNA powder (Sigma-Aldrich, St. Louis, MO, USA; Cat#K3375) in 0.025 M NaOH. The pH of the stock solution was adjusted to 7.38–7.42, followed by its further dilution in either hypoxic solution (i.e., for simulated ischemia) or 10% *v*/*v* FBS-containing medium (i.e., for simulated reperfusion) to reach the desired concentration of 64 µM. The SI/R-induced alterations in mitochondrial morphology were examined using fluorescent and electron microscopy. The effects of KYNA treatment on the SI/R-induced functional impairment of the mitochondria and the degree of mitochondrial superoxide production and mitochondrial membrane depolarization were measured, and assessment of the functionality of mitochondrial respiratory complexes was performed.

#### 2.2.3. Modulation of GPR35 Receptor Activity

To achieve GPR35 receptor activation, cardiac cells exposed to SI/R were treated with Zaprinast at a concentration of 100 µM which has been previously demonstrated to achieve proper activation of the GPR35 receptors [25,26]. To provide treatment solutions, a 100 mM Zaprinast stock solution was prepared by dissolving Zaprinast powder (Sigma-Aldrich; St. Louis, MO, USA; Cat#Z0878) in dimethyl sulfoxide (DMSO; Serva, Heidelberg, Germany; Cat#20385.01), which was then further diluted in either hypoxic solution or 10% FBS-containing medium to reach the desired concentration of 100 µM. To test whether GPR35 receptor inhibition diminishes the KYNA-derived cytoprotection, the cells undergoing SI/R received CID2745687 at a concentration of 1 µM. First, a 10 mM stock solution was provided by dissolving CID2745687 powder (Tocris Bioscience, Bristol, UK; Cat#4293) in DMSO. The stock solution was then further diluted in either hypoxic solution or 10% FBS-containing medium to achieve the desired treatment concentration of 1 µM. The DMSO content of our treatment solutions was kept under 0.2% *v*/*v* to avoid potential cardioprotective effects. The final DMSO concentrations of the solutions applied were 0.1 and 0.002% *v*/*v* for Zaprinast and CID2745687, respectively.

To test whether the inhibition of GPR35 receptor activity prevents the KYNA-induced cytoprotection in the setting of SI/R, combined treatments (i.e., 64 µM KYNA administration in parallel with 1 µM CID2745687 treatment) were provided.

### 2.3. Electron Microscopy

To prepare samples for electron microscopic calcium level assessments and mitochondrial morphometry assessments, cardiac cells were collected from 75 cm^2^ tissue culture flasks immediately after SI/R using 0.25% Trypsin–EDTA (Corning, Budapest, Hungary; Cat#25-053-Cl) for 5 min at 37 °C. The cells were then centrifuged (5 min, room temperature (RT), 400× *g*), and washed in PBS, followed by another centrifugation step (5 min, RT, 400× *g*). Pellets were fixed using 3% glutaraldehyde (Polysciences Inc., Warrington, UK; Cat#111-30-8) supplemented with 90 mM of potassium oxalate (pH 7.40) overnight at 4 °C and rinsed in 7.5% sucrose (Molar Chemicals Kft., Halásztelek, Hungary; Cat#02200) solution containing 90 mM potassium oxalate. Postfixation was performed via incubation in 2% potassium pyroantimonate (Merck, Burlington, MA, USA; Cat#12208-13-8) supplemented with 1% osmic acid (Sigma Aldrich; St. Louis, MO, USA; Cat#201030) for 2 h at 4 °C. Samples were then rinsed in distilled water for 10 min, followed by dehydration using an ascending series of ethanol (from 50% to 100%) and treatment with propylene oxide. Finally, the samples were embedded into Durcupan ACM epoxy resin (Sigma Aldrich; St. Louis, MO, USA; Cat#44611) and polymerized for 48 h at 56 °C. Semithin (0.5 µM) sections were prepared using an Ultracut UCT ultramicrotome (Leica Microsystems, Wetzlar, Germany). Ultrathin (50 nm) sections were then cut and mounted onto single-hole copper grids (Electron Microscopy Sciences; Hatfield, PA, USA), contrasted with 2% uranyl acetate (Electron Microscopy Sciences) in 50% ethanol and 2% lead citrate in distilled water. The ultrathin sections were examined in a JEM-1400Flash transmission electron microscope (JEOL, Tokyo, Japan). Sections were screened at low magnification (2–4000×), and 10–15 randomly selected cells were located. From each cell, images from randomly selected cytoplasmic regions were recorded as 16-bit grayscale images at 10,000× magnification using a Matataki Flash (JEOL) 2 k × 2 k high-sensitivity scientific complementary metal-oxide-semiconductor camera. As a result of the applied sample preparation method, electron-dense deposits (EDDs) were present due to the pyroantimonate-induced precipitation of intracellular calcium ions. The relative volume of mitochondrial EDDs was determined using point counting methods [27,28]. Mitochondrial morphometry assessments in the electron microscopic images was performed using Image-Pro Plus software (version 6.0; Media Cybernetics, Rockville, MD, USA). Each mitochondrion (average of 40 mitochondria per sample) was segmented, and morphometric parameters such as the average area of the mitochondria, Feret’s diameter, aspect ratio, circularity, and perimeter were measured [29,30,31]. The data is expressed as a ratio relative to the corresponding normoxic values.

### 2.4. Observation of Mitochondrial Morphology and Distribution by Mitotracking

To analyze the potential effects of KYNA on the SI/R-induced alterations in mitochondrial architecture, cells were seeded onto glass coverslips at a density of 1.5 × 10^4^ cells/well, grown in 24-well plates for two days, and subjected to SI/R with or without 64 µM KYNA treatment. Normoxic samples were prepared as controls. Mitochondria in the living H9c2 cells were labeled using MitoTracker Deep Red (Thermo Fisher Scientific, Waltham, MA, USA; Cat#M22426) immediately after the SI/R protocol (1 µM, 30 min, 37 °C), followed by fixation of the cells using 4% paraformaldehyde (PFA; Alfa Aesar, Haverhill, MA, USA; Cat#30525-89-4) for 20 min at RT. Nuclei were labeled using Hoechst (Cell Signaling Technology, Danvers, MA, USA; Cat#4082) (1:4000, 10 min, RT). Coverslips were mounted onto slides using Mounting Medium, followed by visualization using a NIKON Eclipse Ti-E/80i fluorescent microscope (Nikon Instruments Inc., Melville, NY, USA) using the same exposition time for all samples. Six to nine randomly selected fields of view were captured per sample at 40× magnification. An observer blinded to the experimental conditions scored the cells according to whether they exhibit aggregated mitochondrial fragments [32]. Cells showing uncertain morphology or imperfect staining were excluded from the analysis. The number of cells displaying a damaged mitochondrial architecture was expressed as a percentage of the total cell count and compared to the corresponding control groups.

### 2.5. RNA Isolation and Polymerase Chain Reaction (qPCR)

To assess the expression of genes encoding proteins driving mitochondrial fusion and fission, total RNA was isolated from H9c2 cells grown in 75 cm^2^ tissue culture flasks and exposed to SI/R with or without 64 µM KYNA using a Monarch^®^ Total RNA Miniprep Kit according to the manufacturer’s instructions (New England BioLabs, Ipswich, MA, USA; Cat#T2010S). RNA was transcribed into cDNA using the Maxima First Strand cDNA Synthesis Kit for RT-qPCR (Thermo Fisher Scientific; Waltham, MA, USA, Cat#K1642). The Luminaris Color HiGreen Low ROX qPCR Master Mix (Thermo Fisher Scientific; Waltham, MA, USA, Cat#K0373) was applied to perform RT-PCR. Sample amplification was performed under the following conditions: 10 min at 95 °C and then 40 cycles of 95 °C for 15 sec and 60 °C for 1 min. Relative transcript levels were determined using 18S rRNA as the reference gene. The applied primers are listed in Table 1.

### 2.6. Western Blotting

Western blot was performed to measure the expression of proteins driving fusion and fission of the mitochondria in cells undergoing SI/R with or without KYNA, as well as to detect the effects of the GPR35 receptor agonist Zaprinast on the expression and activation of caspase-3 in the cardiac cells subjected to SI/R. Sample preparation and protein concentration measurements were performed as described previously [24]. Proteins were separated in 10% polyacrylamide gels, followed by blotting onto 0.45 µM pore size polyvinylidene fluoride membranes (60–90 min, RT), which were then cut horizontally with respect to the molecular weights of the target proteins. Unspecific binding was blocked using 5% *v*/*v* BSA (Serva; Heidelberg, Germany; Cat#11920.06) for 1 h at RT. Membranes were then incubated with specific primary antibodies against the target proteins listed in Table 2, followed by incubation with a horseradish-peroxidase-conjugated Goat Anti-Rabbit secondary antibody (DAKO, Agilent, Santa Clara, CA, USA; Cat#P0048, RRID:AB_2617138, 120 min, RT). Membranes were detected using LumiGlo chemiluminescent reagent (Cell Signaling Technology; Danvers, MA, USA, Cat#7003, 5 min, RT) and exposed to X-ray films. All films were scanned (8-bit, 400 dpi), and the density of each protein band was quantified using Quantity One software (Version 4.6.7, RRID:SCR_014280, Bio-Rad company, Hercules, CA, USA). The protein expression was normalized to α-tubulin expression.

### 2.7. Measurement of Mitochondria-Derived Superoxide Levels

To determine whether KYNA influences the mitochondrial oxidative stress induced by SI/R, mitochondrial superoxide production was detected using MitoSOX (Thermo Fisher Scientific; Waltham, MA, USA; Cat#M36008) staining in living H9c2 cells. Cells were subjected to SI/R with or without 64 µM KYNA treatment, followed by washing and incubation with 2.5 μM MitoSOX-containing DMEM (15 min, 37 °C). The cells were then washed again and covered with mounting medium. MitoSOX-stained mitochondria in the living cells were visualized using a NIKON Eclipse Ti-E/80i fluorescent microscope (Nikon Instruments Inc., Melville, NY, USA), applying the same exposition time for all samples. The fluorescence of MitoSOX was quantified by measuring the fluorescence intensity of 10 randomly selected cells from each visualized field of view (6–9 per sample) using Image J software (version 2; National Institutes of Health, Bethesda, MD, USA).

### 2.8. Estimation of Mitochondrial Membrane Potential Using JC-1 Staining

To determine whether KYNA administration improves SI/R-induced alterations in the mitochondrial membrane potential, JC-1 staining was performed on living H9c2 cells, as described previously [24]. At the end of simulated reperfusion, the cells were washed and incubated with DMEM supplemented with 10 μg/mL JC-1 dye (Thermo Fisher Scientific; Waltham, MA, USA; Cat#M34152) for 10 min at 37 °C. The cells were then washed and covered with 10% *v*/*v* FBS-containing DMEM. JC-1-stained cells were visualized using an Olympus BX51 (Olympus corporation, Shinjuku, Tokyo, Japan) microscope equipped with an Olympus DP70 camera using the same exposition time for all samples. The red-to-green fluorescence intensity ratio, representing mitochondrial membrane potential, was determined using Image J software (RRID:SCR_003070).

### 2.9. Assessment of Mitochondrial Respiration

Mitochondrial O_2_ consumption was assessed in the H9c2 cells using high-resolution fluorespirometry (Oxygraph-2k, Oroboros Instruments, Innsbruck, Austria). At the end of SI/R, cells were collected using 0.25% Trypsin–EDTA (Corning; Cat#25-053-Cl; 5 min, 37 °C), and 3 × 10^6^ cells were then centrifuged (5 min, RT, 400× *g*), washed in PBS, resuspended in 2.3 mL Mir05 medium, and gently pipetted into the respiration chamber. When a stable baseline respiration had been reached (ROUTINE respiration without external substrates), the cells were permeabilized with digitonin (20 µg/mL). The complex-I-dependent oxidative phosphorylation capacity (OXPHOS I) was measured in the presence of complex-I-linked substrates (10 mM glutamate and 2 mM malate) and adenylate-diphosphate (ADP) (2.5 mM) [33]. Rotenone (0.5 µM) was used to (a) inhibit complex I and (b) separate the complex-II-dependent oxidative phosphorylation capacity (OXPHOS II) in the presence of succinate (10 mM) and adenylate. After the inhibition of complex III (antimycin A; 2.5 µM), the respiratory activity of complex IV was measured using ascorbate (2 mM) and the artificial substrate TMPD (0.5 mM). Ascorbate was administered before TMPD to avoid uncontrollable autoxidation of the electron donor. Sodium azide (NaN_3_; 100 mM) was finally added to block complex-IV-linked mitochondrial O_2_ consumption [34]. All measurements were performed under continuous stirring at 37 °C. DatLab software (Version 7.4, Oroboros Instruments, Innsbruck, Austria) was used for online display and respirometry data acquisition and analysis. The respiratory substrates and inhibitors were purchased from Sigma Aldrich. Data were illustrated as the O_2_ flow per cell and expressed in pmol/(s × million cells).

### 2.10. Determination of Metabolic Cell Phenotype Using the Seahorse Analyzer

A Seahorse XFp Analyzer (Agilent Technologies, Santa Clara, CA, USA) was used to determine the metabolic phenotype of the H9c2 cells subjected to the vehicle or 64 µM KYNA treatment. Seahorse XF Cell Energy Phenotype tests were used, which can measure parameters representing mitochondrial respiration and glycolysis in living cells. The assays were performed according to the manufacturer’s instructions. Briefly, H9c2 cells were plated into Seahorse cell culture plates one day before measurement at a density of 7 × 10^3^ cells/well in cell culture medium supplemented with either 64 µM KYNA or the vehicle. One hour prior to the assay, the medium was replaced with Seahorse assay medium (Seahorse XF Base Medium supplemented with 2 mM glutamine and 10 mM glucose; pH was adjusted to 7.4). The treatments were maintained for 24 h altogether. Oligomycin (10 µM) and carbonyl-cyanide-4-trifluoromethoxy-phenylhydrazone (FCCP, 15 µM) containing a stressor mix was used. FCCP is an uncoupling agent allowing proton-transport through the mitochondrial membrane. The cellular oxygen consumption rate (OCR) and extracellular acidification rate (ECAR) was measured. OCR is an indicator of the rate of mitochondrial respiration, while ECAR gives information about the rate of glycolysis. Measurements were repeated three times in triplicate.

### 2.11. Measurement of Cell Viability

To assess the effects of GPR35 receptor activation in the setting of cardiac I/R in vitro, the viability of cells undergoing SI/R with or without Zaprinast treatment was measured using a calcein assay. For this, the cells were incubated with 0.5 µM calcein-AM (Thermo Fisher Scientific; Waltham, MA, USA, Cat#C1430) dissolved in Dulbecco’s Phosphate-Buffered Saline (Sigma-Aldrich; St. Louis, MO, USA; Cat# D8537) for 30 min at 37 °C. The fluorescence intensity was then measured at 490/520 nm using a microplate reader (ClarioStar Plus/Fluostar Optima, BMG Labtech, Ortenberg, Germany). Viability was expressed as a percentage of the average viability detected in the normoxic control group.

### 2.12. Statistical Analysis

The data is expressed as the mean ± standard error of the mean (SEM). The normality of the sample distribution was assessed using the Shapiro–Wilk normality test. For a normal distribution, comparisons involving more than two groups were assessed using a one-way ANOVA using Prism 8.0 software (GraphPad Software, GraphPad Software Ldt., San Diego, CA, USA). The post hoc analysis was performed using either Fisher’ LSD test (i.e., when the sample size was less than 10) or Tukey’s multiple comparisons test (i.e., when the sample size exceeded 10). For non-normally distributed datasets, the Kruskal–Wallis test was applied with Dunn’s multiple comparisons test as the post hoc analysis.

## 3. Results

### 3.1. KYNA Treatment Reduced the Rate of Intramitochondrial Calcium Accumulation and Improved Mitochondrial Ultrastructure in Cells Exposed to SI/R

**Figure 1:**
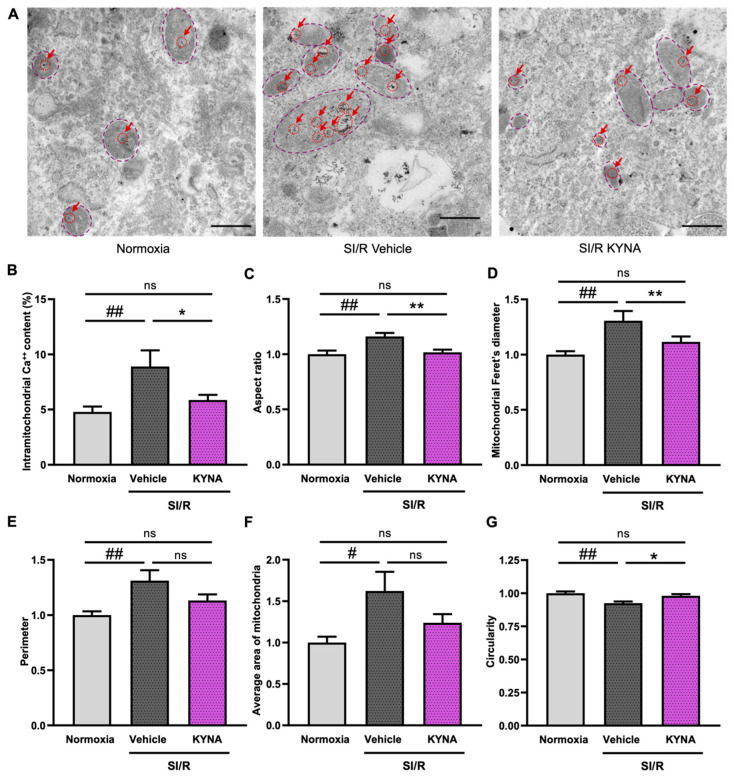
KYNA administration reduced the SI/R-induced increase in mitochondrial calcium levels and alterations in the mitochondrial ultrastructure. (**A**) Representative pictures of electron microscopic analysis (scale bar: 500 nm). Red arrows indicate intramitochondrial calcium deposits. Dashed purple lines outline the extent of individual mitochondria. (**B**) KYNA treatment diminished the SI/R-induced significant increase in the mitochondrial calcium content (10–15 images per sample, *n* = 6 from 3 independent experiments). Mitochondrial morphometry was performed via the quantification of (**C**) aspect ratio, (**D**) mitochondrial Feret’s diameter, (**E**) perimeter (**F**) average area of the mitochondria, and (**G**) circularity (10–15 images per sample, *n* = 10 from 5 independent experiments). For mitochondrial morphometry, individual values were normalized to the average values detected in corresponding normoxic controls. Values were expressed as the mean ± SEM and compared to normoxic or SI/R+vehicle control groups, ## *p* < 0.01 and # *p* < 0.05 vs. normoxia, ** *p* < 0.01 and * *p* < 0.05 vs. SI/R+vehicle, ns: not significant; one-way ANOVA. The post hoc analysis was performed using either Tukey’s multiple comparisons test (i.e., morphometry) or Fisher’s LSD post hoc test (i.e., calcium content measurement).

As calcium overload is a key event in the mechanism of I/R-triggered mitochondrial impairment [35], we measured the average intramitochondrial Ca^2+^ content in electron microscopic images captured from H9c2 cardiac cells undergoing SI/R (i.e., 6 h of simulated ischemia followed by 2 h of simulated reperfusion) with or without KYNA treatment applied throughout SI/R at the previously determined protective dose of 64 µM. Here, SI/R caused a significant increase in mitochondrial calcium levels; KYNA treatment, however, substantially reduced this effect and retained the intramitochondrial calcium levels similar to those under normoxic conditions (Figure 1A,B). To quantify the structural alterations at an ultrastructural level, various parameters, including the aspect ratio (i.e., the ratio of the major and minor axes of a mitochondrion), mitochondrial Feret’s diameter (i.e., the longest distance between any two points of the external perimeter), perimeter, the average area of the mitochondria, and circularity, were measured in the electron microscopic images. The aspect ratio (Figure 1C), Feret’s diameter (Figure 1D), perimeter (Figure 1E), and average area (Figure 1F) were shown to increase significantly upon SI/R, while circularity decreased substantially in the cells undergoing SI/R (Figure 1G). KYNA treatment was shown to revert the SI/R-induced impairments in mitochondrial morphology, as treated cells exhibited a significantly decreased aspect ratio (Figure 1C). Furthermore, KYNA was shown to prevent the SI/R-induced substantial increase in Feret’s diameter, perimeter, and average area (Figure 1D–F), as well as to retain a circularity close to that observed in cardiac cells kept under normoxic conditions (Figure 1G).

### 3.2. Treatment with KYNA Seemed to Prevent the SI/R-Induced Alterations in the Mitochondrial Network

**Figure 2:**
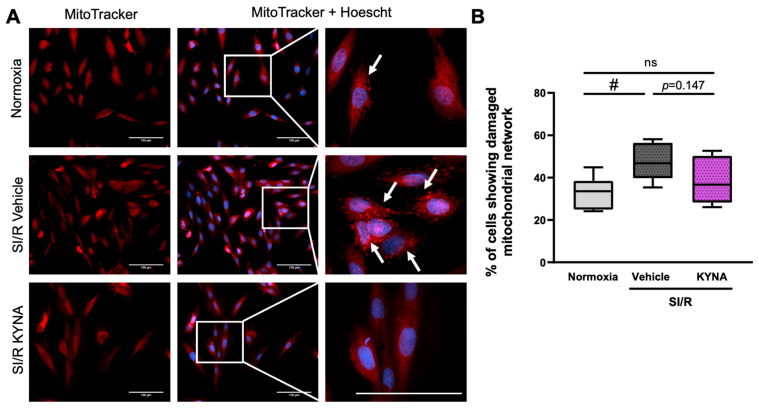
KYNA treatment prevented the SI/R-induced elevation in the ratio of cardiac cells exhibiting an aggregate-like mitochondrial morphology. (**A**) Representative pictures of MitoTracker staining. Scale bar: 100 μm. White arrows indicate an aggregate-like staining pattern. (**B**) Percentage of cells showing altered mitochondrial morphology (*n* = 5–6 samples from 4 independent experiments, 6–8 fields of view/sample). Data were expressed as mean ± SEM, # *p* < 0.05 vs. normoxia, ns: not significant; one-way ANOVA, Fisher’s LSD post hoc test.

Since structural and subsequent functional impairment of the mitochondria is thought to be a key contributor to cardiac I/R injury, we performed MitoTracker staining to detect the potential SI/R-induced alterations in the architecture of the mitochondrial network, as well as to examine whether KYNA treatment affected these changes (Figure 2A). Here, SI/R was shown to disrupt the intracellular organization of the mitochondria compared to that observed in normoxic control groups (i.e., a homogeneous distribution with a mild perinuclear density). Upon SI/R, we observed a strong, aggregate-like mitochondrial staining pattern, suggesting the accumulation of potentially damaged mitochondrial fragments (Figure 2A). Scoring of the cells according to whether they exhibited aggregate-like mitochondrial structures revealed that SI/R increased the ratio of cells showing such morphological alterations significantly (Figure 2A,B). KYNA treatment, however, was found to prevent the SI/R-triggered substantial elevation in the percentage of cells containing aggregated mitochondrial fragments, retaining a ratio closer to that observed in the corresponding control group (Figure 2A,B).

### 3.3. Administration of KYNA Upregulated the Transcription of Mitochondrial Fusion- and Fission-Related Genes, Without Altering Protein Expression Levels

**Figure 3:**
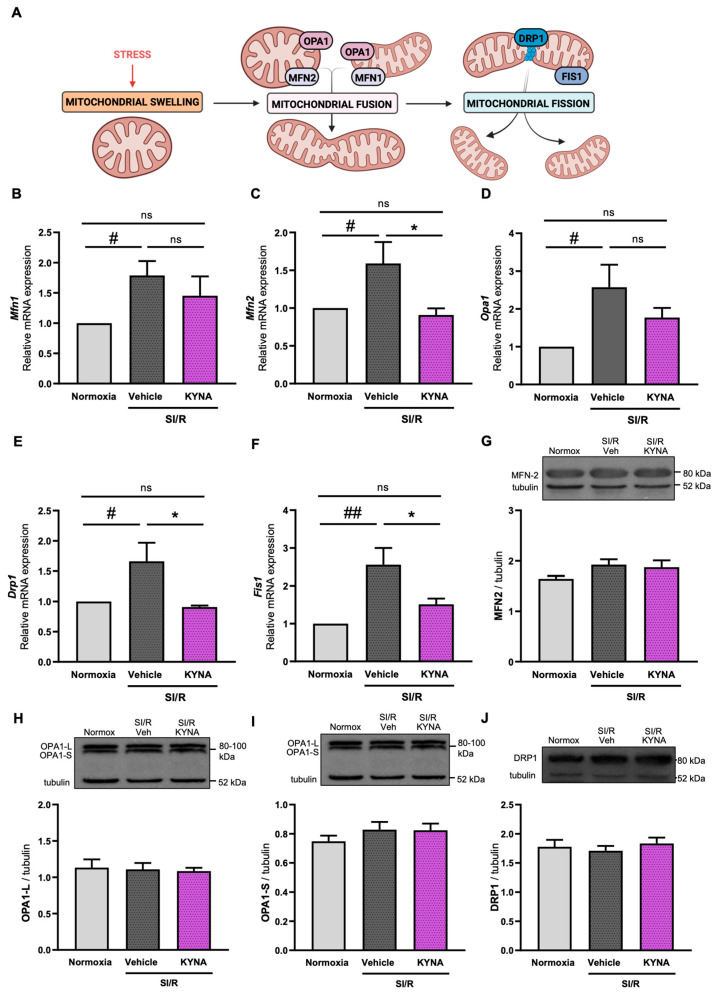
KYNA-derived effects on the expression of fusion- and fission-related genes in cells exposed to SI/R. (**A**) Signaling pathways driving mitochondrial fusion and fission were examined. The SI/R-induced increase in the relative mRNA expression of the (**B**) *Mfn1*, (**C**) *Mfn2*, and (**D**) *Opa1* genes was reduced or prevented by KYNA treatment (*n* = 7–8 from 3 independent experiments). KYNA was shown to diminish the SI/R-induced increase in the relative mRNA expression of the (**E**) *Drp1* and (**F**) *Fis1* genes as well (*n* = 7–8 samples/group from 3 independent experiments). Western blotting revealed no significant changes in (**G**) MFN2, (**H**,**I**) OPA1-L and -S or (**J**) DRP1 protein levels (*n* = 9–13/group, samples were collected from 3–4 independent experiments). The data were expressed as the mean ± SEM and were compared to normoxia and SI/R+vehicle control groups, ## *p* < 0.01 and # *p* < 0.05 vs. normoxia, * *p* < 0.05 vs. SI/R+vehicle, ns: not significant; one-way ANOVA. Post hoc analysis was performed using either Fisher’s LSD post hoc test (i.e., for less powered datasets) or Tukey’s multiple comparisons test. The figure demonstrating the fusion and fission pathways was created using BioRender.com. Original Western blot images for Figure 3G–J can be found in Supplementary Figure S1.

As we found evidence suggesting that both SI/R and KYNA treatment affect the organization of the mitochondrial network, we performed molecular investigations examining whether altered mitochondrial quality control mechanisms lay behind the observed changes in the structure and organization of the mitochondria (Figure 3A). First, we assessed the activation of fusion-driving genes, such as *Mfn1*, *Mfn2*, and *Opa1*. SI/R was shown to stimulate the transcription of *Mfn1*, *Mfn2*, and *Opa1* genes significantly (Figure 3B–D). KYNA treatment, however, was shown to either significantly reduce (i.e., in the case of *Mfn2*) or prevent (i.e., in the case of *Mfn1* and *Opa1*) the SI/R-induced increase in the transcription of profusion genes (Figure 3B–D). Next, the activity of genes encoding fission-driving DRP1 and FIS1 proteins was examined, revealing that the transcription of both *Fis1* and *Drp1* genes increased significantly upon SI/R (Figure 3E,F). KYNA treatment was shown to ameliorate the SI/R-triggered transcription of both investigated genes, significantly reducing *Fis1* and *Drp1* mRNA levels (Figure 3E,F). To confirm the SI/R- and KYNA-induced alterations in fusion and fission mechanisms, we measured the protein-level expression of MFN2, OPA1, and DRP1 (Figure 3G–J). Interestingly, Western blotting revealed no significant SI/R- or KYNA-induced changes in the expression of MFN2 (Figure 3G), OPA1 (Figure 3H,I), or DRP1 (Figure 3J).

### 3.4. KYNA Administration Reduced the Mitochondrial Superoxide Production and Preserved the Mitochondrial Membrane Potential in Cardiac Cells Exposed to SI/R

**Figure 4:**
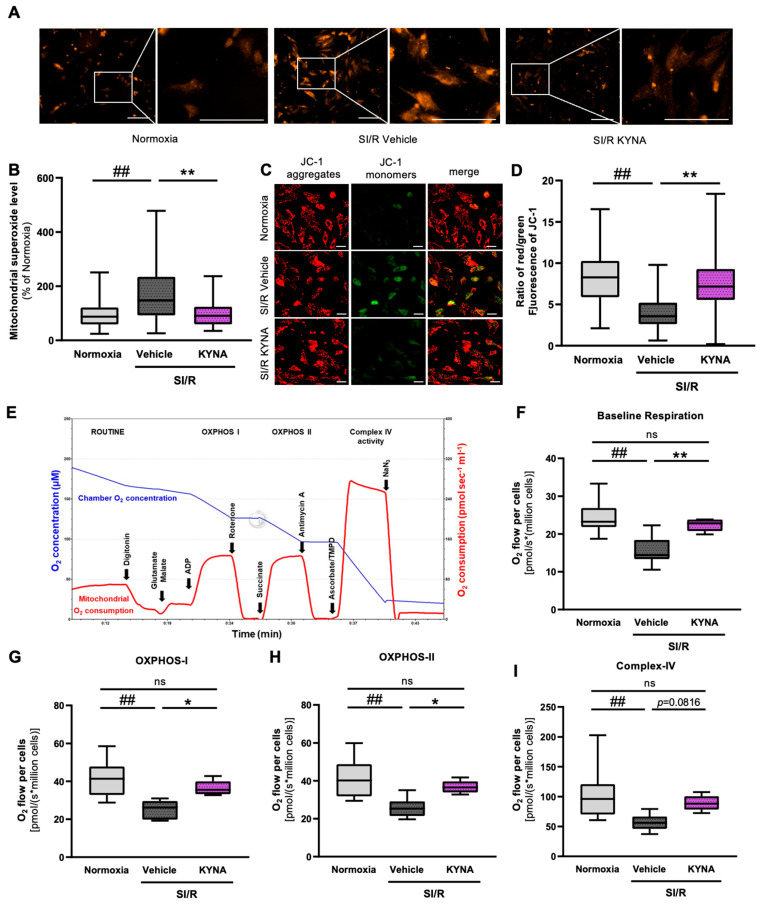
KYNA treatment preserved the functional integrity of the mitochondria in cardiac cells undergoing SI/R. (**A**) Representative pictures of MitoSOX staining. Scale bar: 500 μm. (**B**) KYNA treatment attenuated the SI/R-induced mitochondrial ROS release (fluorescent intensity was measured in 179–194 cells in images captured from randomly selected fields of view; *n* = 2–3/experiment; 4 separated experiments). (**C**,**D**) The depolarization of the mitochondrial membranes upon SI/R was prevented by KYNA (150 cells/each group, *n* = 6 from 3 independent experiments). Scale bar: 500 μm (**E**) The respirometry protocol. (**F**–**I**) KYNA treatment was found to improve all examined parameters of mitochondrial respiration (*n* = 5–8 from 6 independent experiments). The data were expressed as the mean ± SEM and were compared to normoxia and SI/R+vehicle control groups, ## *p* < 0.01 vs. normoxia, ** *p* < 0.01 and * *p* < 0.05 vs. SI/R+vehicle. ns: not significant. Datasets showing a normal distribution were tested using a one-way ANOVA with Fisher’s LSD post hoc test. Datasets showing a non-normal distribution (i.e., superoxide production and mitochondrial membrane potential measurement) were analyzed using the Kruskal–Wallis test followed by Dunn’s multiple comparisons test for post hoc analysis.

To elucidate the potential antioxidant effect of KYNA in the SI/R-induced death of cardiomyocytes, the cells were subjected to SI/R with or without KYNA treatment, followed by the measurement of mitochondrial superoxide levels using MitoSOX staining. Mitochondrial superoxide levels increased substantially after SI/R (Figure 4A,B). Nevertheless, KYNA treatment was found to prevent the SI/R-induced oxidative stress by reducing the excessive mitochondrial production of superoxide significantly (Figure 4A,B). To confirm the mitoprotective feature of KYNA further, SI/R-induced alterations in the mitochondrial membrane potential were assessed. Staining the cell cultures with a mitochondrial-membrane-potential-sensitive dye (i.e., JC-1) revealed that SI/R resulted in the depolarization of the mitochondrial membrane (i.e., decreased the mitochondrial accumulation of the red-fluorescent JC-1 aggregates, in parallel with an increased level of the green-fluorescent monomer form) (Figure 4C,D). Importantly, KYNA treatment protected the mitochondria from SI/R-induced depolarization, retaining the mitochondrial membrane potential close to that observed in normoxic controls (Figure 4C,D).

### 3.5. KYNA Treatment Improved Parameters of Mitochondrial Respiration in Cardiac Cells Undergoing SI/R

As we showed that KYNA may preserve mitochondrial functions in H9c2 cardiac cells undergoing SI/R, mitochondrial respiration was investigated using high-resolution respirometry (Figure 4E) to support this hypothesis further. Here, SI/R caused a considerable decrease in baseline respiration as compared to that in the normoxic controls (Figure 4F); however, KYNA treatment markedly attenuated this SI/R-triggered effect, retaining baseline O_2_ consumption values similar to those observed in the normoxic group (Figure 4F). In all groups, plasma membrane permeabilization with digitonin resulted in a rapid decline in baseline ROUTINE respiration due to the diffusion of endogenous substrates out of the cell [36]. However, respiration increased again when saturating concentrations of substrates and ADP were added into the chambers (Figure 4E). In comparison to the normoxic group, SI/R was shown to impair complex-I- and complex-II-dependent oxidative phosphorylation (OXPHOS I, -II), as well as complex-IV-linked respiration (OXPHOS IV) (Figure 4G–I). The administration of KYNA restored (i.e., in the case of OXPHOS I and II) or prevented (i.e., in the case of OXPHOS IV) the SI/R-induced deterioration in mitochondrial respiration, as the oxygen consumption rates were shown to increase in the KYNA-treated cells in all respiratory states compared to those observed in the vehicle-treated cells undergoing SI/R (Figure 4G–I). In a separate experiment, KYNA did not influence cellular respiration in the absence of SI/R as assessed using the Seahorse Analyzer (Figure S2A–C). These data indicate that KYNA might elicit a beneficial impact on mitochondrial respiration in the setting of SI/R.

### 3.6. The Cardiocytoprotective Effect of KYNA Involves GPR35 Receptor Activation

**Figure 5:**
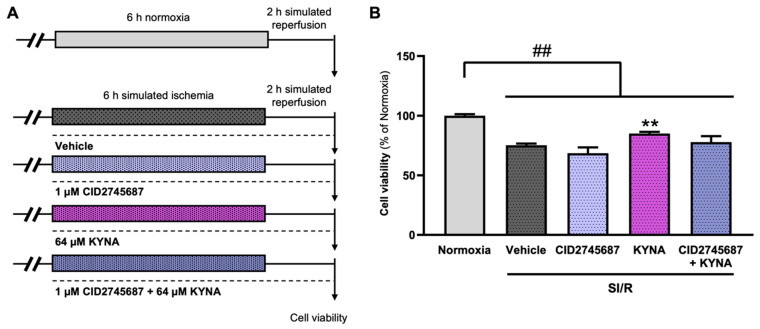
Stimulation of GPR35 receptors was involved in KYNA-mediated cardiocytoprotection. (**A**) The experimental protocol. (**B**) The KYNA-derived cytoprotection seemed to diminish in the presence of GPR35 antagonism. The data were expressed as the mean ± SEM and were compared to normoxia, SI/R+vehicle, and SI/R CID2745687 groups, ## *p* < 0.01 vs. normoxia, ** *p* < 0.01 vs. SI/R+vehicle. Data were tested using the Kruskal–Wallis test with Dunn’s multiple comparisons test as the post hoc analysis.

Next, we aimed to examine whether KYNA-derived cytoprotection relies on the activation of the GPR35 receptors. For this, combined treatments with KYNA and CID2745687, a synthetic GPR35 receptor antagonist, were administered throughout SI/R (Figure 5A). As observed before, SI/R reduced the survival of the H9c2 cells significantly (Figure 5B), while KYNA treatment, applied at the previously uncovered protective dose of 64 µM, improved the cell viability substantially (Figure 5B). The GPR35 receptor antagonist CID2745687 was administered at a concentration of 1 µM, which has been shown to exert no significant effect on cell viability in the setting of SI/R (Figure 5B). Although KYNA treatment alone rescued cells undergoing SI/R, here, we found that when administered in parallel with CID2745687, the KYNA-derived protective effect diminished (Figure 5B).

### 3.7. GPR35 Receptor Activation by Zaprinast Improved the Survival and Mitochondrial Function of Cardiac Cells Subjected to SI/R

**Figure 6:**
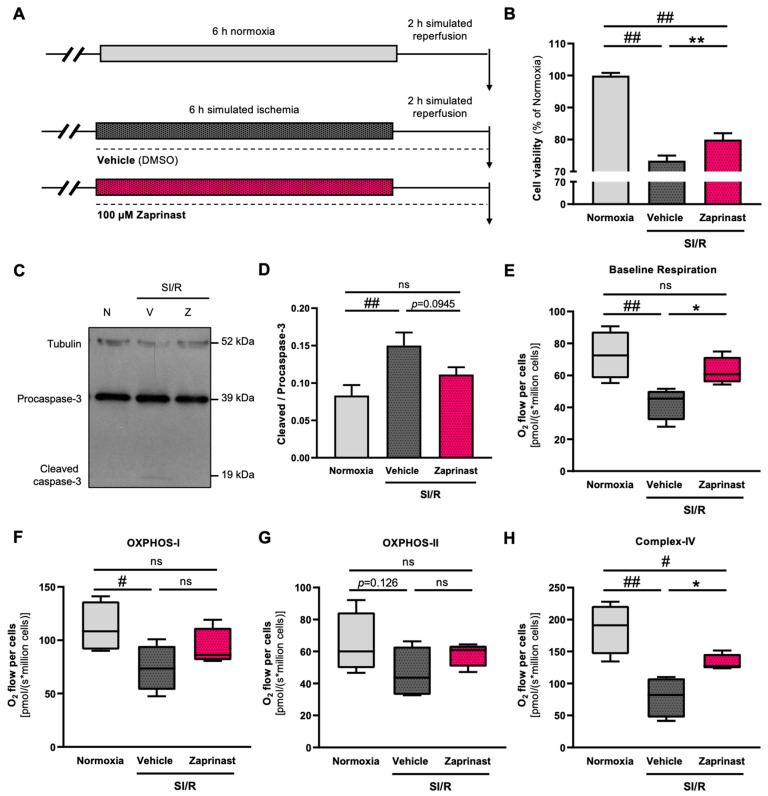
Stimulation of GPR35 receptor activity using Zaprinast improved the survival of cardiac cells undergoing SI/R and preserved mitochondrial function. (**A**) The experimental protocol. (**B**) Zaprinast treatment improved the survival of cardiac cells exposed to SI/R (*n* = 14–28/experiment, 5 independent experiments). (**C**,**D**) Zaprinast decreased the SI/R-induced activation of caspase-3 (*n* = 6–8 from 2 independent experiments). Original Western blot images for Figure 6C can be found in Supplementary Figure S1. (**E**–**H**) High-resolution fluorespirometry revealed that Zaprinast retained baseline respiration and did not seem to affect complex I- and -II-dependent but improved complex-IV-dependent oxidative phosphorylation (*n* = 4 from 4 independent experiments). Data were expressed as the mean ± SEM and were compared to normoxia and SI/R+vehicle control groups, ## *p* < 0.01 and # *p* < 0.05 vs. normoxia, * *p* < 0.05 and ** *p* < 0.01 vs. SI/R+vehicle, ns: not significant. The datasets showing a non-normal distribution (i.e., viability measurements) were tested using the Kruskal–Wallis test with Dunn’s multiple comparisons test as the post hoc analysis. For a normal data distribution (i.e., Western blot and high-resolution respirometry), one-way ANOVA was applied using Fisher’s LSD post hoc test.

As the KYNA-derived increase in the survival of cardiac cells undergoing SI/R is most likely to occur due to receptor-mediated mechanisms, next, we assessed whether GPR35 receptor activation induced cardiocytoprotection in the present experimental setting after we confirmed the expression of such receptors on the H9c2 cells (Supplementary Figure S3). To stimulate the GPR35 receptors exclusively (i.e., without the modulation of other KYNA target receptors), Zaprinast was administered throughout the 8 h of the SI/R protocol at a concentration of 100 µM (Figure 6A). Here, GPR35 receptor activation increased the survival of cardiac cells exposed to SI/R significantly (Figure 6B). To corroborate these findings, caspase-3 activation was analyzed via Western blotting, revealing that SI/R increased caspase-3 activation significantly (Figure 6C,D). Zaprinast administration, however, seemed to prevent such a great increase in the rate of procaspase-3 cleavage and activation, retaining cleaved/procaspase-3 levels closer to those found in the normoxic controls (Figure 6C,D). To assess whether the modulation of the GPR35 receptors exclusively impacted mitochondrial function as well, high-resolution respirometry was performed. As previously, SI/R caused a significant decrease in baseline respiration compared to that observed in the normoxic controls (Figure 6E). Cardiac cells receiving Zaprinast treatment during SI/R showed substantially higher baseline respiration rates compared to those in the vehicle-treated cells (Figure 6E). SI/R was shown to reduce complex-I- and -IV-dependent OXPHOS significantly, while complex-II-linked respiration exhibited a tendentious decrease upon SI/R (Figure 6F–H). Zaprinast treatment reduced the degree of the SI/R-induced alterations in OXPHOS-I and -II and was found to alleviate the effect of SI/R on complex-IV-linked respiration significantly (Figure 6F–H).

## 4. Discussion

In the present study, we demonstrated that the KYNA-derived cardiocytoprotection involves mitoprotective features, such as the maintenance of a healthy mitochondrial morphology and the prevention of mitochondrial ROS release, as well as the preservation of mitochondrial function, that seem to be evoked at least in part due to GPR35 receptor agonism. Although numerous investigations have been carried out to develop novel treatment strategies preserving the functionality of myocardial tissue exposed to I/R, currently, there is no effective and clinically available therapy to reduce I/R-induced damage of the cardiac tissue. Kynurenic acid has previously been shown to exert protection against I/R-induced cardiac cell damage in multiple preclinical settings [22,23,37,38]; however, its precise mechanism of action has not been fully clarified yet.

Mitochondrial Ca^2+^ overload is a key event in I/R-triggered mitochondrial impairment [39], initiating a sequence of harmful effects affecting mitochondrial functions (e.g., the stimulation of mitochondrial production of free radicals, as well as mitochondrial permeability transition pore opening and consequent depolarization), eventually culminating in cytochrome-C release and the initiation of intrinsic apoptosis [40,41]. Our results demonstrated that exposure of the cardiac cells to SI/R substantially increased intramitochondrial Ca^2+^ levels, while KYNA treatment was shown to reduce the mitochondrial Ca^2+^ content in cells undergoing SI/R. This is in line with previous findings showing KYNA-induced antiapoptotic features [23], which may be due to reduced mitochondrial Ca^2+^ overload. Further supporting the beneficial role of KYNA in the regulation of Ca^2+^ homeostasis, it has previously been demonstrated that KYNA administration suppresses an NMDA-induced increase in intracellular Ca^2+^ levels in cultured astrocytes expressing GluN1-subunit-containing NMDA receptors [42]. As Ca^2+^ overload promotes prolonged opening of the mitochondrial permeability transition pores, leading to mitochondrial swelling, ultimately resulting in rupture of the mitochondria [43,44], next, we examined the potential ultrastructural changes induced by SI/R or KYNA treatment at the single-mitochondrion level via electron microscopy. Our results demonstrated that the mitochondria in the cells undergoing SI/R exhibited a significantly increased aspect ratio, Feret’s diameter, perimeter, and average area, in parallel with decreased circularity, suggestive of swelling and elongation of the mitochondria [29,45]. KYNA treatment, however, was shown to maintain the proper morphology of the mitochondria in cells undergoing SI/R. Organization of the mitochondria was visualized using MitoTracking to further corroborate that KYNA impacts SI/R-induced alterations in the arrangement of the mitochondria. Here, KYNA administration was shown to reduce the ratio of cells exhibiting aggregate-like structures in their mitochondrial network. Mitochondrial quality control mechanisms, consisting of multiple coordinated pathways such as fusion and fission of the mitochondria, determine the organization of the mitochondrial network and are fundamental to maintaining cellular mitochondrial homeostasis [46,47,48]. Physiological fusion is an adaptive mechanism repairing damaged mitochondrial fragments by joining them to neighboring intact mitochondria, thereby enabling the exchange of matrix constituents [33]. It is generally followed by fission, which recreates normal, functioning mitochondria, contributing to the rearrangement of the mitochondrial network [33]. Uncontrolled fusion and fission, however, are important contributors to various pathologies [49]. Over-fission of the mitochondria, for example, promotes mtDNA damage, reducing the transcription of genes encoding respiratory complex components, which eventually leads to decreased mitochondrial respiration, depleted energy production, and increased formation of mitochondrial ROS [50]. Furthermore, over-enhanced fission prolongs mPTP opening, thereby contributing to the induction of intrinsic apoptosis [50]. Our results demonstrated that the transcription of genes encoding both profusion and fission-driving mediators was upregulated in the cardiac cells undergoing SI/R, an effect which was diminished upon KYNA treatment. However, these changes were not yet detectable at the level of protein expression. Although we have not investigated this phenomenon further, it seems plausible that altered mitochondrial quality control mechanisms did not contribute to the observed alterations in mitochondrial structure and function in our experimental setup. However, increased mRNA levels may support the idea that fusion and fission contribute to late-onset injury in the cardiac cells following I/R, which could be prevented by KYNA administration. Other mechanisms underlying the unaffected protein levels (e.g., potential post-transcriptional regulatory mechanisms attenuating the effects of SI/R and KYNA on the expression of fusion- and fission-driving proteins) remain to be investigated.

Recent discoveries have demonstrated that alterations in mitochondrial shape and architecture correlate with both cellular energy status and mitochondrial functions [51,52,53]. Mitochondrial-impairment-induced enhancement of mitochondrial ROS production has been described to be a fundamental contributor to AMI-induced damage of the cardiac cells [4,47]. Previous studies have reported KYNA-derived antioxidant effects in the neuronal and hepatic tissues [13,21]; moreover, we have shown that KYNA treatment attenuates the SI/R-caused increase in cellular superoxide levels [23]. To characterize the antioxidant effects of KYNA in the setting of SI/R further, we determined the level of mitochondrial ROS production in cardiac cells undergoing SI/R and assessed the potential antioxidant effect of KYNA. Here, we found significantly increased mitochondrial superoxide levels after SI/R, which were found to diminish upon KYNA administration. These results are in accordance with recent observations in neonatal mouse cardiomyocyte cultures subjected to SI/R [37]. Furthermore, it has been described that KYNA administration prevents the I/R-induced decrease in the expression of superoxide dismutase 2 enzyme [38]. Oxidative stress has been shown to contribute to the collapse of the mitochondrial membrane potential, which is a key event in AMI-induced damage of the cardiac cells [54,55]. Here, we have shown that KYNA treatment protected the mitochondria from the SI/R-induced loss of the mitochondrial membrane potential. This observation is in line with recent findings reporting similar KYNA-derived effects using a different approach in another H9c2-cell-based model of myocardial I/R [38]. Besides membrane potential measurements, assessment of mitochondrial respiration is another key parameter of mitochondrial function. Interestingly, contradictory results are available regarding the effects of KYNA on ischemia-induced energy depletion. On the one hand, it has been shown that exogenous KYNA administration (≥125 µmol/L) to mitochondria isolated from cardiac cells might impair mitochondrial function and lower ATP production [56]. In addition, it has been demonstrated that neither I/R nor KYNA application alters the expression of respiratory chain complex proteins and their activities [38]. On the other hand, KYNA was shown to prevent ischemia-induced ATP depletion via activation of the GPR35 receptors in IPS-derived cardiomyocytes exposed to I/R [37]. Keeping these contradictory reports in mind, we determined the effects of exogenously administered KYNA on mitochondrial respiration using high-resolution respirometry. Our results demonstrated that KYNA applied during SI/R improved the baseline oxygen consumption, as well as reverting the SI/R-induced decreases in complex-I-, -II- and -IV-linked respiration. Moreover, it caused significantly higher O_2_ consumption rates in all of the examined respiratory states (OXPHOS I, OXPHOS II, and complex-IV-linked respiration) compared to those in the corresponding vehicle-treated groups. Beneficial effects of KYNA on mitochondrial respiration have been demonstrated in a rat model of sepsis-associated mitochondrial impairment in the nervous tissue as well, further corroborating that KYNA might prevent cellular demise through the preservation of respiratory chain complex activities [57]. Although our investigations have been carried out on H9c2 cells, others have reported somewhat similar KYNA-derived protective effects (e.g., decreased cell death or infarct size, decreased oxidative stress) observed in primary cultures and iPSC cells, as well as in ex vivo and in vivo circumstances [22,37,38], further corroborating the potential role of KYNA in cardioprotection.

KYNA-derived effects are most likely to occur through the modulation of receptor-mediated intracellular pathways. KYNA is known to block ionotropic glutamate receptors (e.g., NMDA and AMPA receptors), as well as to stimulate the activity of the AhR and GPR35 receptors. Although the widely investigated KYNA-derived neuroprotective features have mostly been attributed to the metabolite’s NMDA-receptor-antagonistic properties [12,58], previously, we have shown that blocking NMDA receptors with either KYNA or another, synthetic antagonist, MK-801, does not impact the survival of cardiac cells undergoing SI/R significantly [23]. Among further KYNA target receptors, GPR35 receptors have been reported to exert promising effects considering potential cardioprotective mechanisms. For example, Wyant et al. recently demonstrated that KYNA seems to prevent ischemia-induced ATP loss through GPR35 receptor activation [37]. Additionally, GPR35 receptor agonism has been found to exert protection against ischemic injuries of the central nervous system too [59]. However, controversial observations have been made, with reports that the suppression of GPR35 protects the heart from ischemic injury via reducing oxidative stress and mitochondria-dependent intrinsic apoptosis [60]. Furthermore, it has been reported that GPR35 stimulation increases ROS generation [61]. To address these contradictory findings, in the present study, we investigated the significance of GPR35 receptor agonism in KYNA-induced cardiocytoprotection. Firstly, to confirm that GPR35 agonism is necessary for KYNA-derived cytoprotection, we assessed whether KYNA protected the cardiac cells when applied in parallel with CID2745687, a well-known GPR35 receptor antagonist. Although the beneficial KYNA-derived effects were observed when it was administered alone, we found that inhibition of GPR35 receptor activity via simultaneous treatment with CID2745687 diminished the KYNA-induced cytoprotection in our experimental setup. The effects of GPR35 receptor activation using another agonist, Zaprinast, were then investigated, revealing that stimulation of the GPR35 receptors significantly improved the survival of the cardiac cells exposed to SI/R (Table 3). Furthermore, this treatment seemed to prevent the SI/R-induced substantial increase in apoptotic activity, as revealed through the detection of active cleaved caspase-3 protein levels. In addition, we have shown that Zaprinast-induced GPR35 receptor agonism might preserve the functionality of the mitochondria, similarly to KYNA, suggesting that KYNA-derived mitoprotection might occur at least in part due to GPR35 receptor stimulation. These findings suggest that activation of the GPR35 receptors during I/R leads to protective effects that are similar to those exerted by KYNA treatment (Table 3). Taken together, our results suggest that GPR35 receptor agonism seems to play an important role in KYNA-mediated protection against SI/R-induced damage to the cardiac cells. This is in line with recent observations demonstrating that KYNA-derived protection against ischemic myocardial injury is mediated through the GPR35-receptor-activation-induced maintenance of ATP production [37]. However, as the Zaprinast treatment was found to induce less pronounced protective effects in our experimental setup, it is likely that the KYNA-driven protection against I/R injury involves further molecular mechanisms besides those initiated by GPR35 receptor activation. For example, antagonism of the AMPA receptors using inhibitors other than KYNA has been reported achieve protection against myocardial I/R injuries [62]; hence, AMPA receptors are another promising candidate which might be involved in the conduction of KYNA-triggered protection.

In summary, here, we have demonstrated that KYNA-derived protection against I/R-induced cardiac cell injury involves various mitoprotective features, preserving both the structure and function of the mitochondria. Our results have also implicated that GPR35 receptor agonism elicits similar effects and is involved in the mechanism of KYNA-mediated cytoprotection.

### 4.1. Future Perspectives and Translational Considerations

The future development of cardioprotective drugs based on the beneficial mechanisms triggered by KYNA requires further investigations—for example, with regards to the additional molecular pathways involved or confirmation of their efficiency in other, optionally animal models. As for safety, previously, we showed that KYNA administration under stress-free conditions does not impact cell viability [23], suggesting its safe applicability. This idea is supported by the fact that various cells have been reported to tolerate even higher KYNA concentrations [63,64]. Although systemic KYNA administration has not been tested on humans, a phase I, randomized, double-blind clinical trial supported the safety and tolerability of topically delivered KYNA [65]. Additionally, certain food (e.g., chestnut honey), the consumption of which has not been associated with adverse effects, may contain high amounts of KYNA, suggesting translational value for dietary supplementation [66]. However, its pharmacokinetics after systemic administration and optimal routes of delivery (e.g., the administration of exogenous KYNA or the stimulation of endogenous KYNA release via altering the activities of enzymes governing the kynurenine pathway) remain to be investigated in the future. The safety and effectivity of KYNA analogues, other promising candidates for drug development, are also yet to be analyzed. One of these, namely AV-101 (i.e., 4-chlorokynurenine), was tested in a phase I, randomized, double-blind, placebo-controlled, crossover clinical study and was found to be safe and well tolerated [67]. Nevertheless, as a natural, endogenously synthesized compound, KYNA seems to be a promising candidate for the future development of cardioprotective drugs.

### 4.2. Limitations of the Study

Although we have provided several lines of evidence to reveal the underlying mechanism of action of KYNA against I/R-induced myocardial injuries, our work is not without limitations. Firstly, our results rely on in vitro-based experiments using cardiomyoblasts. Therefore, our findings are limited to demonstrating some additional aspects of the KYNA-derived beneficial effects that may arise through the protection of either differentiated cardiomyocytes or other cell types found in the myocardium. Nonetheless, some of our results have been corroborated by others using ex vivo and in vivo settings. Secondly, additional investigations are required to clarify whether further KYNA target receptors (i.e., AhR, AMPA, or kainate receptors) are involved in the KYNA-mediated protection of cardiac cells. Thirdly, although our study focused on the effects of KYNA on some aspects of mitochondrial quality control, the potential role of further, either survival or cell death, pathways, such as autophagy, ferroptosis, pyroptosis, or endoplasmic reticulum stress, remains to be investigated. Additionally, we have provided evidence that KYNA applied at 64 μm triggers mitoprotection when administered throughout the period of I/R. Other setups, involving either pre- or post-I/R treatment regimens, as well as the mitochondria-related effects of KYNA when it is applied at other concentrations should be tested in the future to identify the most sufficient type of application. Also, it should be examined further whether KYNA exerts any significant effects on mitochondrial structure and function under normoxic conditions. Nevertheless, we have provided novel details on the mechanism of action of KYNA-derived cardiocytoprotection in the setting of myocardial I/R, while future studies are needed to clarify the molecular action of KYNA against cardiac I/R injury further.

## 5. Conclusions

In the present study, we have demonstrated that KYNA exerts mitoprotective effects in the setting of SI/R that involve the maintenance of physiological intramitochondrial Ca^2+^ levels, preservation of the structural integrity of the mitochondria, a reduction in mitochondrial oxidative stress, and protection against the loss of the mitochondrial membrane potential, as well as working against impairment of mitochondrial respiration. Furthermore, we have reported that the survival and mitochondrial respiration of cardiac cells treated with Zaprinast (i.e., another GPR35 receptor agonist) improved significantly. On the other hand, antagonism of GPR35 receptor activity in parallel with KYNA treatment diminished the KYNA-induced cytoprotection, suggesting that stimulation of GPR35-receptor-mediated pathways plays a fundamental role in KYNA-derived protection. As mitochondrial impairment is considered to be a cornerstone of cardiac injury and a driver of I/R-induced pro-apoptotic mechanisms, our results, demonstrating the mitoprotective features of KYNA in the setting of cardiac I/R injury, might contribute to the improvement of novel treatment strategies for AMI patients.

## Figures and Tables

**Table 1:** Primers used for RT-PCR. *Mfn1*: mitofusin-1; *Mfn2*: mitofusin-2; *Opa1*: optic atrophy 1; *Drp1*: dynamin-related protein 1; *Fis1*: mitochondrial fission proteins.

Genes	Forward Primers (5′→3′)	Reverse Primers (5′→3′)
*Mfn1*	GAAGGCCTGTCCAGAACTGA	CCGGGTTCCTGTATGTTGCT
*Mfn2*	CCCTTACCAGCTAGAAACGAGA	GACAAAGTGCTTGAGAGGGGA
*Opa1*	TGCTGTTGGAGGTGGCTATAC	GGTGTACCCGCAGTGAAGAA
*Drp1*	CGTAGTGGGAACTCAGAGCAG	ACCCCATTCTTCTGCTTCAACT
*Fis1*	GGTTGCGTGGTAAGGGATGA	CAAACTGCGTGCTCTTGGAC
*Gpr35*	GCTCTTTGCAGGTTGTGACTG	GCACGGCTGAAGATGTTTCG

**Table 2:** Primary antibodies used for Western blotting. MFN2: mitofusin-2; OPA1: optic atrophy 1; DRP1: dynamin-related protein 1; ON: overnight; CST: cell signaling technology.

Protein	Manufacturer, Catalog No.	Conditions
MFN2	CST, Cat#11925 (RRID: AB_2750893)	1:1000, 4 °C, ON
OPA1	CST, Cat#80471 (RRID: AB_2734117)	1:1000, 4 °C, ON
DRP1	CST, Cat#8570 (RRID: AB_10950498)	1:1000, 4 °C, ON
Caspase-3	CST, Cat#14220 (RRID: AB_2798429)	1:1000, 4 °C, ON
Cleaved caspase-3	CST, Cat#9664 (RRID: AB_2070042)	1:750, 4 °C, ON
α-tubulin	CST, Cat#2114 (RRID: AB_2210548)	1:2000, 4 °C, ON

**Table 3:** A summary of the observed KYNA- and Zaprinast-triggered effects on cell viability and apoptotic activity, as well as mitochondrial structure and function. #: previously reported findings, ↑ increased and ↓: decreased, ns: not significant.

Investigated Parameter	Effects of KYNA on SI/R-Induced Changes	Effects of Zaprinast on SI/R-Induced Changes
Cell viability	↑ #	↑
Apoptotic activity	↓ #	↓
Mitochondrial function:	improved	improved
(1) Baseline respiration	↑	↑
(2) OXPHOS-I	↑	↑ (ns)
(3) OXPHOS-II	↑	↑ (ns)
(4) Complex-IV activity	↑ (ns)	↑

## Data Availability

All of the data supporting the findings of this study are available from the corresponding author on reasonable request.

## Supplementary Materials

The following supporting information can be downloaded at https://www.mdpi.com/article/10.3390/biom15101481/s1, Figure S1: Original Western blot images for Figure 3G–J and Figure 6C; Figure S2: Kynurenic acid (KYNA) has no significant effect on the metabolic phenotype of H9c2 cells under normoxic conditions; Figure S3: GPR35 receptors are expressed on H9c2 cardiomyocytes.

## Abbreviations

The following abbreviations are used in this manuscript:

ADP	adenylate-diphosphate
AhR	aryl hydrocarbon receptor
AMI	acute myocardial infarction
AMPA	α-amino-3-hydroxy-5-methyl-4o-isoxazolepropionic acid
GPR35	G-protein coupled receptor 35
I/R	ischemia/reperfusion
KYNA	kynurenic acid
NMDA	N-methyl-D-aspartic acid
OXPHOS	oxidative phosphorylation
SI/R	simulated ischemia/reperfusion
