# Supplementary material for: Kynurenic Acid Protects Against Myocardial Ischemia/Reperfusion Injury by Activating GPR35 Receptors and Preserving Mitochondrial Structure and Function
Source: Biomolecules. 2025 Oct 21;15(10):1481. doi: 10.3390/biom15101481 (PMC12562769; doi:10.3390/biom15101481)
Supplement: Supplementary file 1 [file biomolecules-15-01481-s001.zip › biomolecules-3874211-supplementary.pdf]

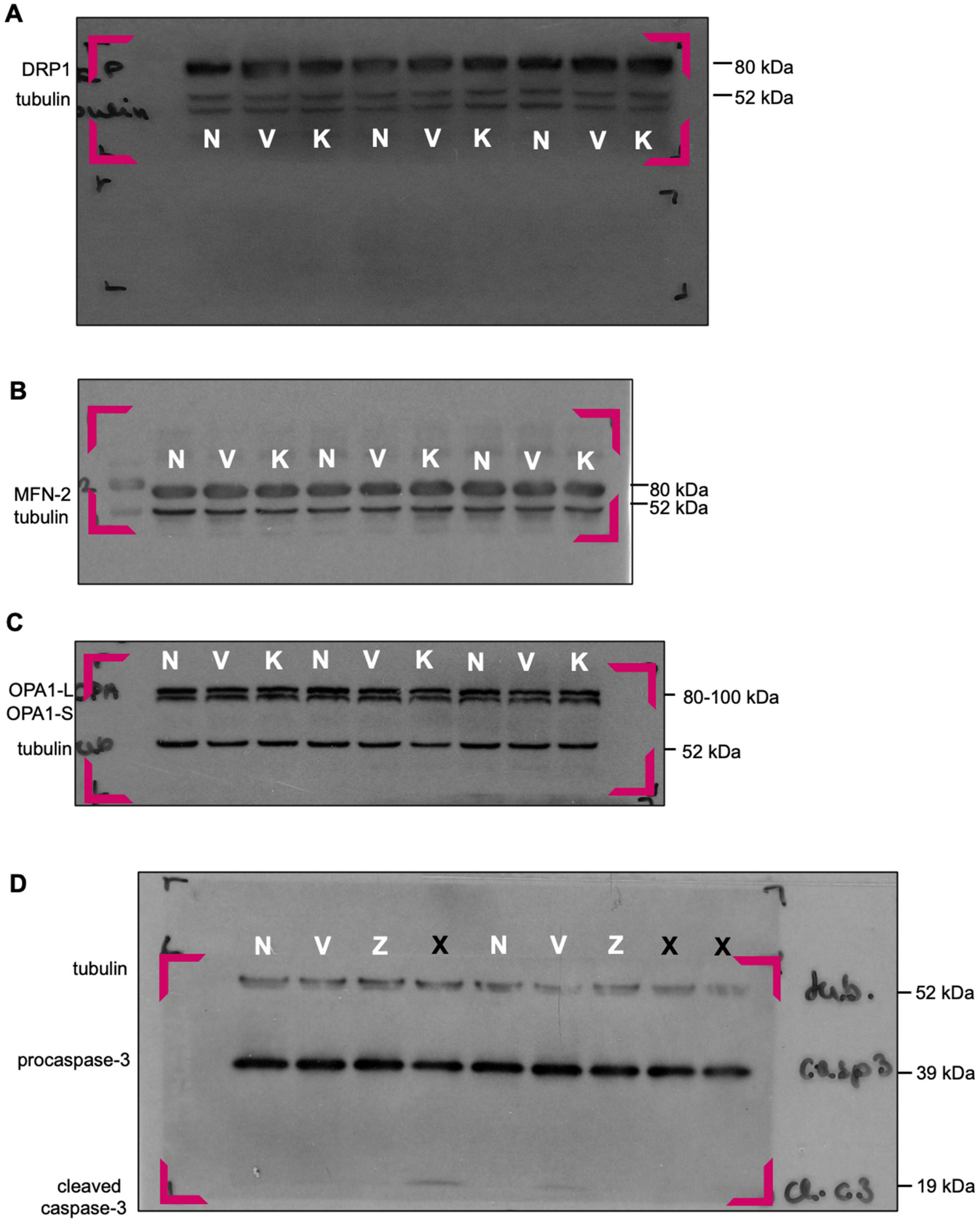

Figure S1. Original Western Blots blot images for Figure 3G–J. Expression (**A**) DRP1, (**B**) MFN2, (**C**) OPA1 proteins. N: Normoxia, V: SI/R + Vehicle (0.05 mM NaOH), K: SI/R + KYNA. (**D**) Expression of active caspase-3. N: Normoxia, V: SI/R+Vehicle (DMSO), Z: SI/R + Zaprinast.

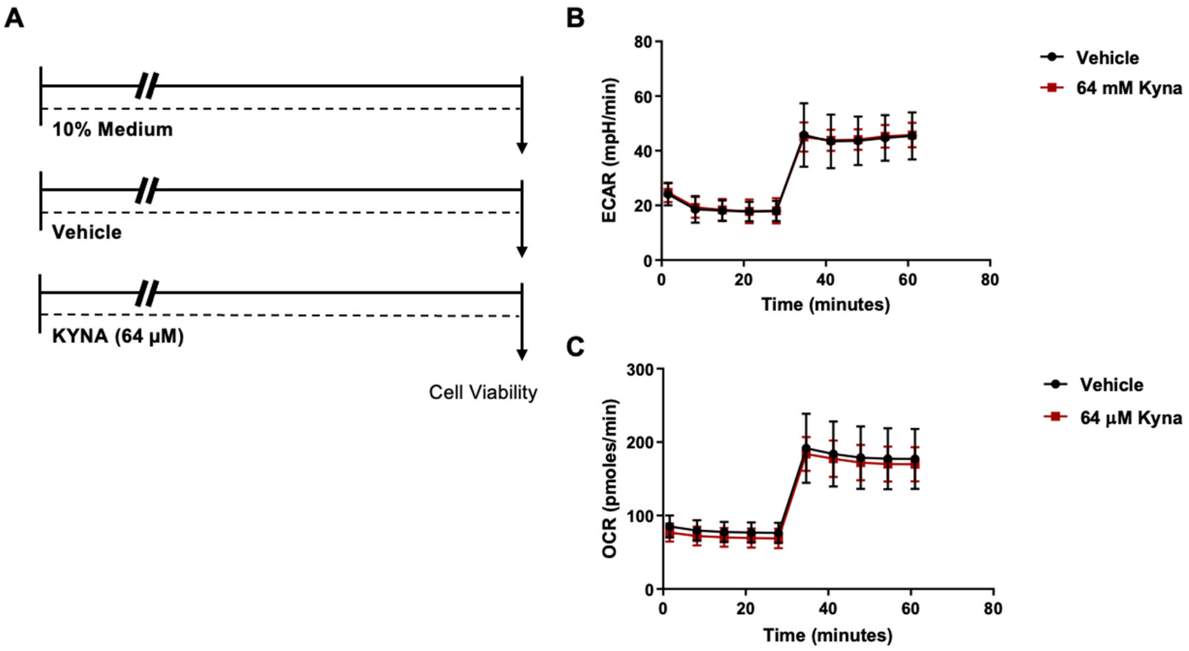

Figure S2. Kynurenic acid (KYNA) has no significant effect on the metabolic phenotype of H9c2 cells under normoxic conditions. (A) H9c2 cardiomyoblasts were treated with 64 µM KYNA under normoxic conditions for 24 h. (B-C) Seahorse analyzer was applied to examine the potential effects of KYNA on the metabolic phenotype of cells characterized by extracellular acidification rate (ECAR) and oxygen consumption rate (OCR) (n=3/experiment, 3 separate experiments). Values are expressed as mean ± S.E.M. and compared to medium or vehicle groups. No significant effects of KYNA were detected on cellular oxidative stress and metabolic phenotype under normoxic condition.

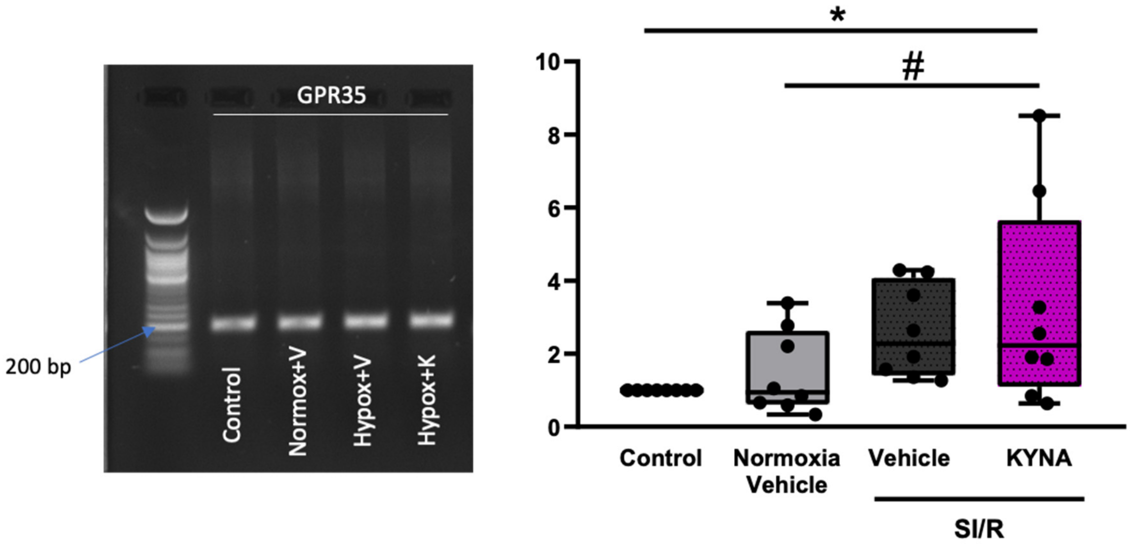

Figure S3. GPR35 receptors are expressed on H9c2 cardiomyocytes. Expression of GPR35 receptors was investigated using quantitative RT-PCR (n=7-8 from 4 independent experiments. Vlues were expressed as mean ± S.D. on box and whiskers plot, Statistical analysis was performed using one-way ANOVA with Fischer’s LSD post hoc test revealing that GPR35 receptor expression increased significantly in the SI/R+KYNA group compared to both Control (i.e., cultures maintained under medium) and Normoxia+Vehicle groups.
